# Nanomedicine-enabled chemotherapy-based synergetic cancer treatments

**DOI:** 10.1186/s12951-021-01181-z

**Published:** 2022-01-04

**Authors:** Wencheng Wu, Yinying Pu, Jianlin Shi

**Affiliations:** 1 The State Key Lab of High Performance Ceramics and Superfine Microstructures, Shanghai Institute of Ceramics, Chinese Academy of Sciences, Shanghai, 200050 People’s Republic of China; 2 Center of Materials Science and Optoelectronics Engineering, University of Chinese Academy of Sciences, Beijing, 100049 People’s Republic of China; 3 Department of Medical Ultrasound, School of Medicine, Shanghai Tenth People’s Hospital, Ultrasound Research and Education Institute, Tongji University, Shanghai, 200072 People’s Republic of China; 4 Platform of Nanomedicine Translation, School of Medicine, Shanghai Tenth People’s Hospital, Tongji University, Shanghai, 200072 People’s Republic of China

**Keywords:** Nanomedicine, Chemotherapy, Synergisitic cancer treatments

## Abstract

Chemotherapy remains one of the most prevailing regimens hitherto in the fight against cancer, but its development has been being suffering from various fatal side effects associated with the non-specific toxicity of common chemical drugs. Advances in biomedical application of nanomedicine have been providing alternative but promising approaches for cancer therapy, by leveraging its excellent intrinsic physicochemical properties to address these critical concerns. In particular, nanomedicine-enabled chemotherapy has been established as a safer and promising therapeutic modality, especially the recently proposed nanocatalytic medicine featuring the capabilities to generate toxic substances by initiating diverse catalytic reactions within the tumor without directly relying on highly toxic but non-selective chemotherapeutic agents. Of special note, under exogenous/endogenous stimulations, nanomedicine can serve as a versatile platform that allows additional therapeutic modalities (photothermal therapy (PTT), photodynamic therapy (PDT), chemodynamic therapy (CDT), etc.) to be seamlessly integrated with chemotherapy for efficacious synergistic treatments of tumors. Here, we comprehensively review and summarize the representative studies of multimodal synergistic cancer treatments derived from nanomedicine and nanocatalytic medicine-enabled chemotherapy in recent years, and their underlying mechanisms are also presented in detail. A number of existing challenges and further perspectives for nanomedicine-synergized chemotherapy for malignant solid tumor treatments are also highlighted for understanding this booming research area as comprehensively as possible.

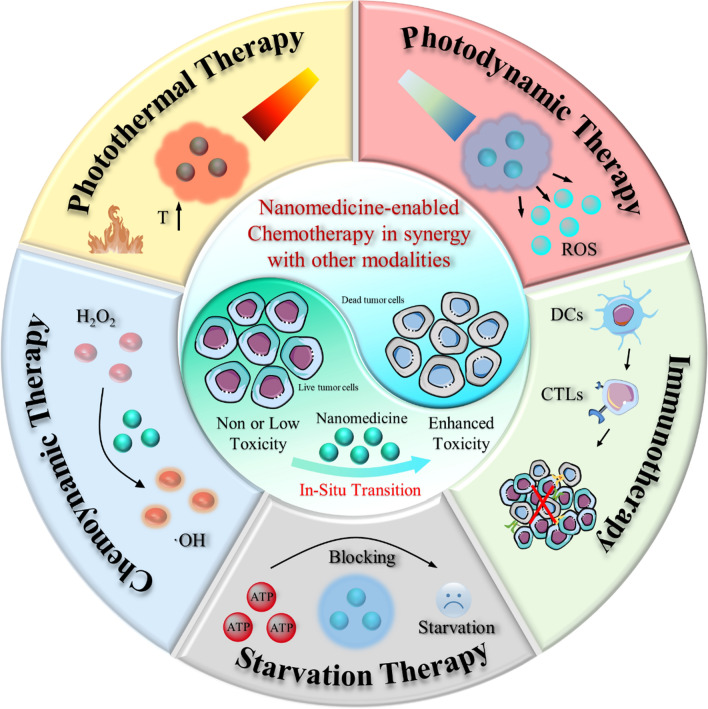

## Introduction

For centuries, cancer has been considered one of the most dangerous diseases seriously threatening human health, and even now, cancer treatment remains one of the major challenges in biomedical application [1]. Chemotherapy has been the standard therapy in clinics for both primary and metastatic cancers for a long time period, which employs cytotoxic chemical drugs to induce the apoptosis or necrosis of tumor cells [2, 3]. Numerous novel chemodrugs have been developed or synthesized for combating cancer since World War II. However, various undesirable side effects and multiple drug resistance (MDR) [4] induced by chemotherapy make cancer treatment highly prone to failure [5]. Also, the harsh tumor microenvironment that hinders chemical drug penetration usually leads to disappointing clinical outcomes. Oncogenic mutations in cancer patients will lead to continual cancerous replications and consequent invasions and metastases to normal tissues, whilst the efficacy of conventional single chemotherapy will be undoubtedly counteracted by the heterogeneity of tumors and the complexity of cancer physiology based on its single treatment modality, in addition to its inevitable harm to normal tissues and organs. Consequently, searching and developing more efficient and safer cancer treatment regimens are undoubted of great research value and clinical benefits.

With the rapid development of nanotechnology, various multifunctional agents at nanoscale can be successfully synthesized, showing great potential as nanomedicines in diagnosing, preventing, and treating disease. Especially in the cancer therapy areas, nanocarrier-based drug delivery systems (NDDSs) have drawn extensive attention. Numbers of nano-formulations have now been approved by the Food and Drug Administration (FDA) for clinical cancer treatment (e.g., paclitaxel albumin-bound nanoparticles (Abraxane) and liposomal irinotecan (Onivyde), etc.), and there are much more numbers of nanomedicines in clinical trials. Unlike molecular chemical drugs without specific targeting ability, engineered nanomedicine can selectively or even on-demand release their cargo upon specific stimulations at the tumor tissues, thus maximizing the therapeutic effect and minimizing toxic side effects [6–8]. More attractively, nanomedicine-mediated in situ conversion of non-toxic/low-toxic substances into cytotoxic therapeutic products within the tumor may be a more sophisticated but appealing avenue to circumvent the adverse effects of conventional chemotherapy.

**Scheme 1:**
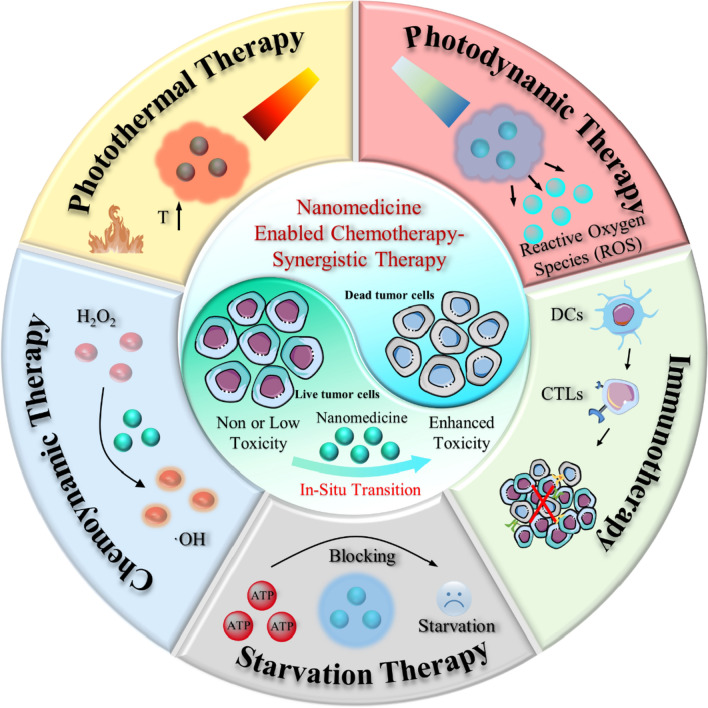
Summary of the nanomedicine-enabled chemotherapy and derived synergistic cancer therapy

Although nanomedicine-enabled chemotherapy has demonstrated the desirable anti-tumor efficacy, the single chemotherapy strategy often fails to eliminate cancer cells, which may lead to cancer recurrence threatening the patient's life and health [9–11]. With the in-depth understanding of tumor biology, molecular pathways, and tumor microenvironment, the current trend in nanomedicine-based chemotherapy has gradually promoted the research focus shift from monotherapy to multimodal synergistic cancer treatment [12–14]. By specifically responding to biological factors (pH, redox potential, and enzymes) within the tumor as well as other exogenous stimuli (light, magnetism, ultrasound, and X-rays), the intelligent nano-platforms can not only serve as transport vehicles for effective drug loading and facilitate their in situ transformation but also seamlessly integrate other therapeutic modalities (photothermal therapy (PTT), photodynamic therapy (PDT), chemodynamic therapy (CDT), etc.) with chemotherapy for pursuing augmented anti-cancer effects [15–18]. Here, we focus on recent advances in nanomedicine-enabled chemotherapy-synergized multimodal cancer treatments (Scheme 1). First, we will elucidate briefly on the rationale, mechanisms, and advantages of nanomedicine-augmented chemotherapy. Then, the positive collaboration between nanomedicine-based chemotherapy and other therapies, such as PTT, PDT, CDT, immunotherapy, and starvation therapy, will be explored comprehensively. Finally, the current challenges and development prospects of this multimodal synergistic treatment based on nanomedicine-enabled chemotherapy were further summarized and analyzed. On the basis of the potential collaborative mechanisms and the design guidance of these intelligent multifunctional nanoagents, it is expected to provide a better understanding of this fast-growing and important area for cancer therapy and facilitate its clinical translation.

## Rational design for nanomedicine-enabled chemotherapy

Given their unique physicochemical properties, nanomedicines hold great promise for cancer therapy, particularly in improving the efficacy and mitigating the adverse effects of conventional chemotherapy [19]. Although heavily debated recently on the enhanced permeability and retention (EPR) effect, nanomedicines have indeed shown to be promising drug delivery systems (DDSs) to specifically deliver elevated dosages of drugs into tumor tissues via EPR effects, thus improving the therapeutic efficacy of tumor chemotherapy and diminishing adverse reactions [20]. By re-programming the intrinsic properties and engineering the surface functions, the NDDSs can be further engineered to become intelligent multifunctional nanoplatforms capable of responding to in vivo or in vitro stimulations. Moreover, the obstruction of the tumor biological barrier and the complex tumor micro-environment, as well as the poor penetration of conventional chemical drug molecules, etc., would usually lead to suboptimal clinical outcomes. Thus far, a growing number of nano-agents have been designed to augment the penetration of therapeutic agents at tumor sites, such as size/surface charge- or particle shape-transformable nanomedicines. [19] It is also noteworthy that P-glycoprotein (P-gp), normally expressed on cell membranes, can pump cytotoxic small molecules out of tumor cells, while nanosized nanomedicines could facilitate the cellular uptake of chemotherapeutic drugs via phagocytosis for bypassing the P-gp-mediated drug efflux and avoiding MDR. And nanomedicine-enabled chemotherapy in combination with P-gp inhibitors could be a reliable strategy for reversing cancer MDR. With its profound biocatalytic performance, nanomedicine also allows the conversion of non-toxic/low-toxic internal substances/delivery substances into cytotoxic products without directly using highly toxic chemical drugs, so as to minimize the side effects on other organs.

## Nanomedicine-enhanced chemotherapy

As the first-line treatment strategy against cancer, chemotherapy has made tremendous achievements in suppressing tumor proliferation, preventing metastasis, and prolonging patients' lives [20]. Commonly used chemodrugs such as doxorubicin (DOX), paclitaxel (PTX), docetaxel (Dtxl), and cisplatin (DDP), etc., can effectively destroy cancer cells after being taken up by tumor cells. However, the therapeutic effectiveness of these chemical drugs is always severely compromised by rapid clearance and non-specific distribution, resulting in inevitable systemic toxicity. Additionally, tumor cells will gradually develop a strong resistance pathway against chemotherapy drugs during long-term administration, known as MDR, which is one of the primary reasons for chemotherapy failure. Nowadays, nanomaterial-enabled chemotherapy aims to elevate the efficacy of conventional cancer chemotherapy regimens through diverse strategies.

### Targeted delivery of chemotherapy drugs

**Fig. 1:**
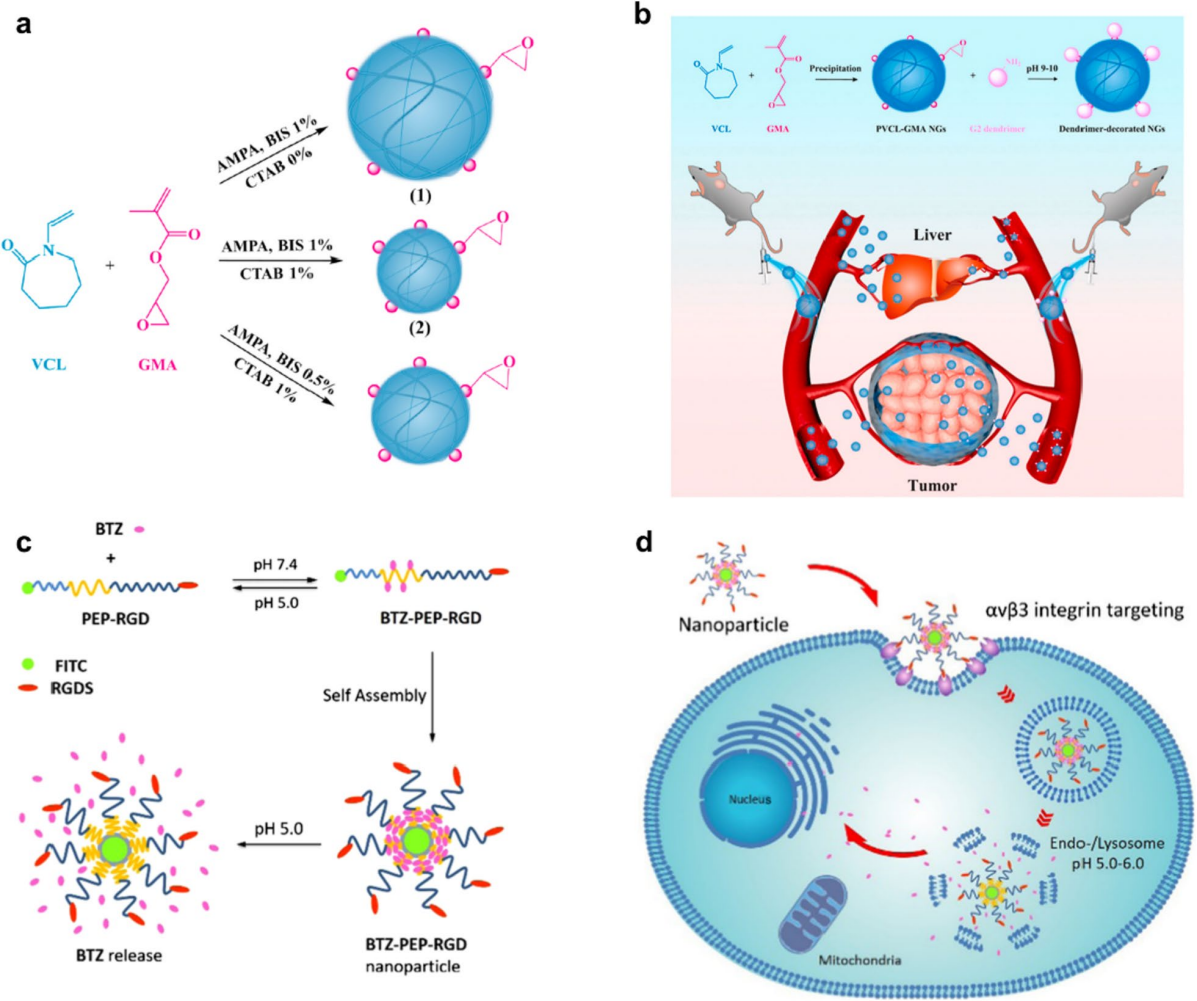
Nanomedicine enhanced chemotherapy through different mechanisms. **a**, **b** Schematic diagram of the fabrication of PVCL-GMA NGs of different diameters and dendrimers decorated PVCL-GMA NGs (NGs-G2) used for efficient drug delivery in vivo. **c**, **d** Schematic representation of pH-responsive drug release and cancer cells targeting behavior of nanomedicine

The enhanced tumor vascular permeability and inhibition of lymphatic drainage allow nanomedicines to enter the interstitial space of the tumor and then be retained, which is the typical EPR effect that achieves passive enrichment of therapeutic agents in the tumor [21]. Numerous studies have been reported in this area. For example, most recently, Li et al. developed a novel cationic dendrimer-decorated nanogels (NGs-G2) to reduce the detrimental retention of chemotherapeutics in the liver while increasing their accumulation in tumors (Fig. 1a) [22]. After modification by the G2 dendrimer, the overall charging NGs-G2 of is turned from neutral into positive, and it exhibits overall charging properties of positively charged corona and neutral core. This unique architecture and charge conversion confers NGs-G2 with highly desired biodistribution of reduced liver accumulation, increased tumor uptake, and facilitated drug release, leading to greatly enhanced anti-tumor therapeutic efficacy with no significant side effects (Fig. 1b). In addition, to further amplify the interactions between nanomedicines and cancer cells, Ma et al. fabricated a novel cancer chemotherapy nanomedicine by self-assembly of the mussel-derived tumor-targeting peptide with a pH-sensitive antitumor drug (Fig. 1c) [23]. In particular, this tumor-targeting peptide RGD enhances the enrichment of chemotherapeutic agents in tumor tissue by binding to αvβ3 integrins overexpressed on tumor cell membranes, making the self-assembled nanoparticles tumor-targeting (Fig. 1d).

### Improving tumor tissue penetration

It is known that the ability of nanomedicines to penetrate tumors may be significantly affected by their physicochemical properties, such as amphipathicity, size, charge as well as shape [24–26]. Over the last few decades, to improve the penetration of chemotherapeutics in tumors, numerous smart nanomedicines have been explored. [27].

**Fig. 2:**
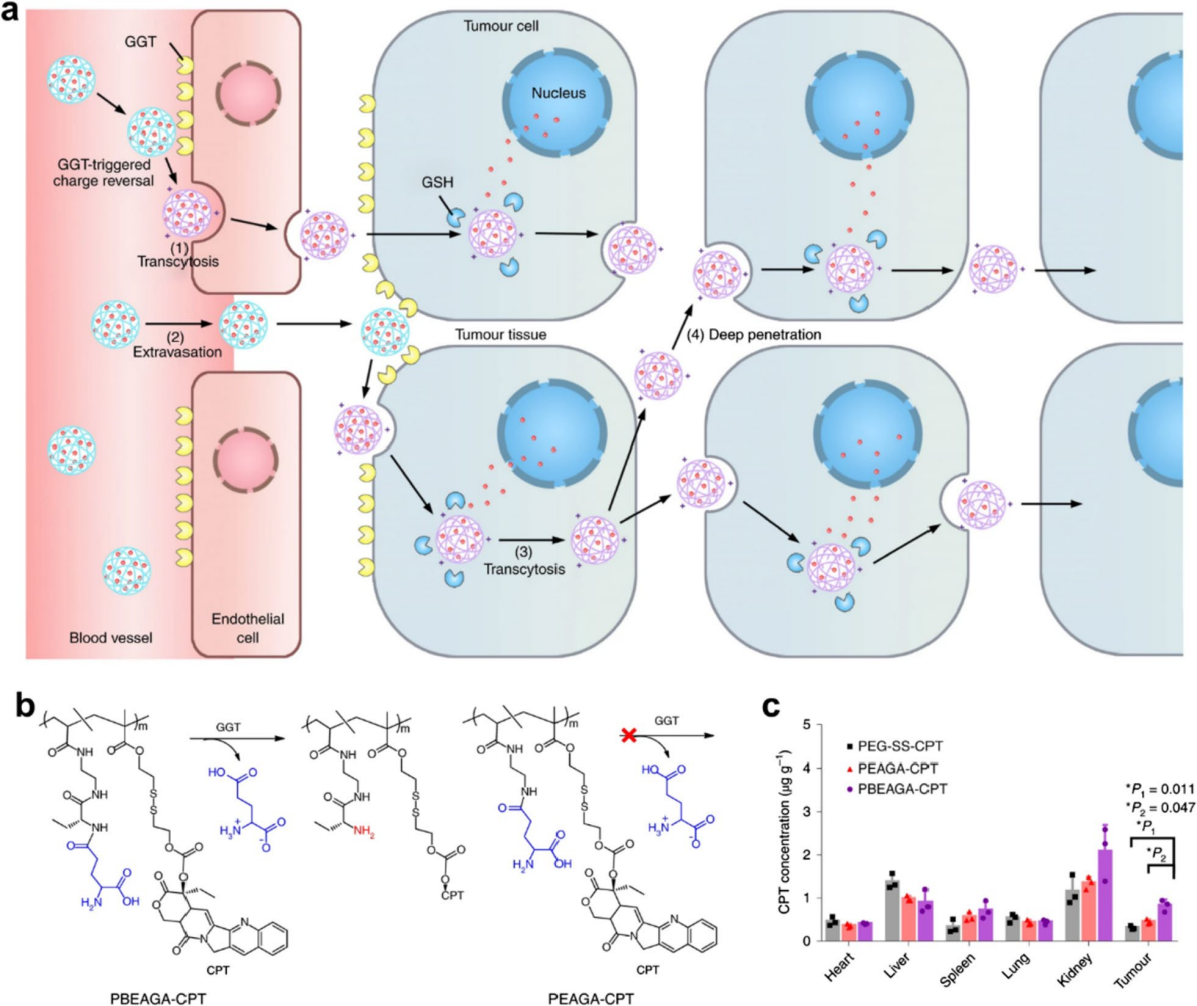
**a** Schematic representation of active tumor penetration of nanomedicine mediated by cationization-initiated transcytosis. **b** The structures with or without GGT-responsive cationized drug-conjugated nanomedicine. **d** The biodistribution of CPT within 24 h after intravenous administration of different nanomedicines

Notably, Shen's team [28, 29] synthesized a series of enzyme-responsive polymer-drug conjugation to improve tumor penetration through active transport by endocytosis and transcytosis, rather than just relying on passive nanoparticle-mediated EPR effects to improve the therapeutic outcomes of chemodrugs. In an innovative study by this group [28], a γ-glutamyl transpeptidase (GGT)-responsive camptothecin-polymer conjugate has been created that could actively penetrate the entire tumor via transcytosis for boosting outcomes of cancer chemotherapy. This nanosized and negatively charged nanomedicine is capable of remaining stable and continuously circulating in the blood compartments. When the nanomedicines passed (PBEAGA-CPT) through the luminal endothelium of tumor vessels or extravasated into the tumor mesenchyme, the γ-glutamyl moieties of the nanomedicines would be split by the γ-glutamyl transpeptidase overexpressed on the cell membrane, producing positively charged primary amines (Fig. 2a).

The generated cationic nanomedicines underwent caveolae-mediated endocytosis and transcytosis, achieving transendothelial and transcellular transport, as well as a relatively uniform distribution in the entire tumor. Compared to the drug-conjugate without GGT-responsive cationized behavior (PEAGA-CPT, Fig. 2b) and the normal long-circulating PEGlated drug conjugate (PEG-SS-CPT), PBEAGA-CPT showed less accumulation in the liver but remarkably more accumulation in the tumor, which contributed to the enhanced chemotherapeutic effect of CPT (Fig. 2c).

### Reversing multidrug resistance

**Fig. 3:**
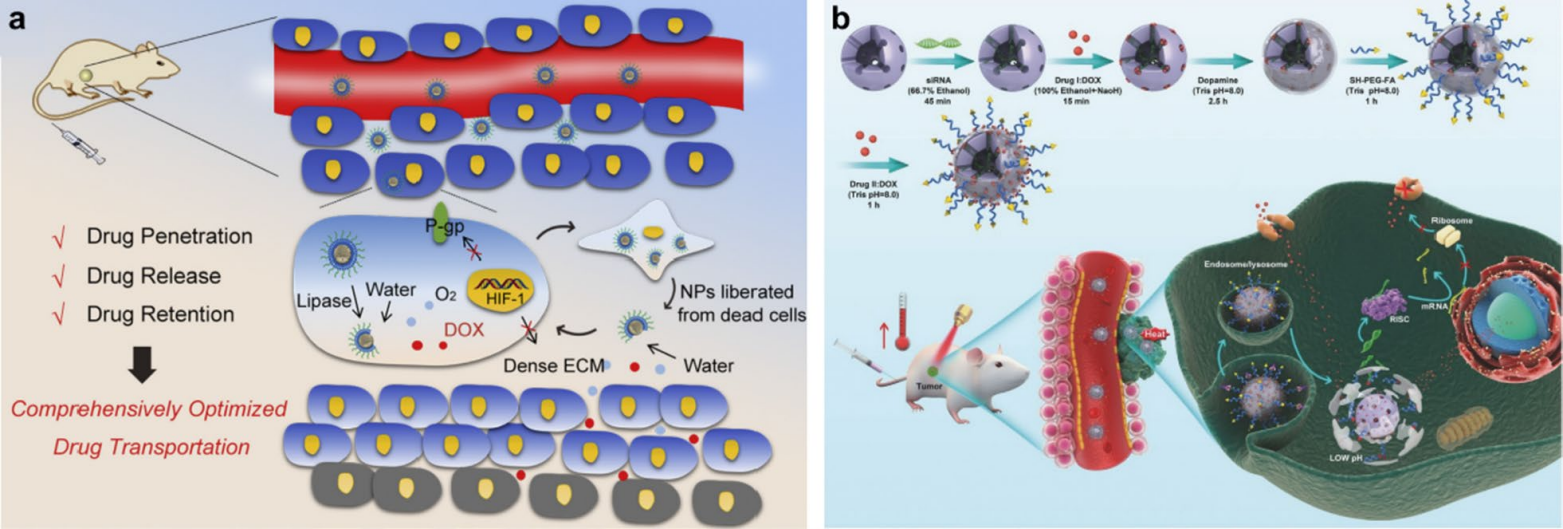
**a** Schematic illustration of DOX-CaO_2_/MnO_2_-MS NPs for largely promoted drug transport. **b** Schematic diagram of the synthesis process of M-R@D-PDA-PEG-FA-D and the combination of photothermal chemotherapy and gene targeting for the treatment of tumors

Despite the important role of nanomedicine-based targeted delivery and strategies to promote tissue penetration in improving cancer chemotherapy, MDR is still a critical issue that cannot be ignored. Based on this background, Zhang et. al. designed and fabricated a solid lipid monostearin (MS)-coated CaO_2_/MnO_2_ vehicle to comprehensively facilitate the DOX transport and be used for overcoming MDR (**Fig. ****3**) [22]. After the nanomedicine was passively enriched in tumor sites via EPR effect and taken up by cancer cells, they would experience the following steps: firstly, the MS layers of NPs were disintegrated by the highly expressed lipase in cancer cells, exposing the core of the active drug. Then, the exposed DOX-CaO_2_/MnO_2_ cores would react with H_2_O, resulting in the explosive release of DOX and O_2_. Finally, O_2_-induced HIF-1/P-gp inhibition conquered the drug pumping-out effect of P-gp, which prolonged intracellular DOX retention time for realizing elevated anticancer activity. In another report, Cheng et al. [30] successfully prepared a multifunctional nanomedicine (M-R@D-PDA-PEG-FA-D) that simultaneously delivers siRNA to downregulate the expression of P-gp protein for enhancing the chemotherapy of DOX (Fig. 3b).

## Nanomedicine-instructed chemotherapy synergistic cancer therapy

Given the significant complexity, diversity, and heterogeneity of neoplasms, nanomedicine-enabled single chemotherapy modality is difficult to achieve satisfactory clinical anti-cancer effects. As such, the current trend in tumor treatment has gradually shifted from single chemotherapy to nanomedicine-enabled synergistic multimodal therapies which have the potential to trigger significant super-additive (namely “1 + 1 > 2”) therapeutic effects due to the synergistic enhancement interactions between chemotherapy and other therapy modalities. In recent years, nanomedicine-enabled synergistic therapies for augmenting cancer chemotherapy have been developed, such as chemotherapy in combination with CDT, PTT, PDT, starvation therapy, and immunotherapy.

### Nanomedicine-mediated synergistic chemotherapy and CDT

The nontoxicity-to-toxicity transition of clinically approved "old" chemodrugs achieved by nanomedicine-triggered chemical reactions in tumor tissue provides a potent strategy for cancer chemotherapy without severe systemic toxicity. Disulfiram (DSF) that has been approved by U. S. Food and Drug Administration (FDA) for the treatment of alcoholism could perform a powerful anti-cancer effect after being converted into bis(N, N-diethyl dithiocarbamate)copper (CuET or CuL_2_) through chelating Cu^2+^. However, the further development of DSF chemotherapy in vivo is severely hampered by the undesirable intrinsic biodistribution of Cu^2+^ in the body and exogenous Cu^2+^ supply caused toxicity [31]. On the bright side, several copper ion-based catalytic reactions might be utilized for treating various diseases by generating therapeutic agents [32]. For instance, CDT, such an emerging tumor therapy modality, could be realized by using the Cu^+^-based Fenton-like reaction to generate ·OH within tumor. Novel nanocatalytic medicines have thus been explored recently for the specific co-delivery of DSF and Cu^2+^ in tumors for CDT synergistic chemotherapy of DSF.

Building on this context, we report a DSF/Cu^2+^ co-delivery nanocatalytic medicine (designated as DSF@PEG/Cu-HMSNs) using hollow mesoporous silica nanoparticles as the functional nanocarrier [33]. After DSF@PEG/Cu-HMSNs enter tumor cells, their framework can rapidly collapse under a mild acidic tumor microenvironment (TME) to quickly co-release Cu^2+^ and loaded DSF. Subsequently, an in situ chelation reaction between co-released Cu^2+^ ions and DSF which produces toxic CuET products, and a Fenton-like reaction between generated Cu^+^ ions and overexpressed H_2_O_2_ in tumor which ROS, proceed simultaneously under acidic TME.

**Fig. 4:**
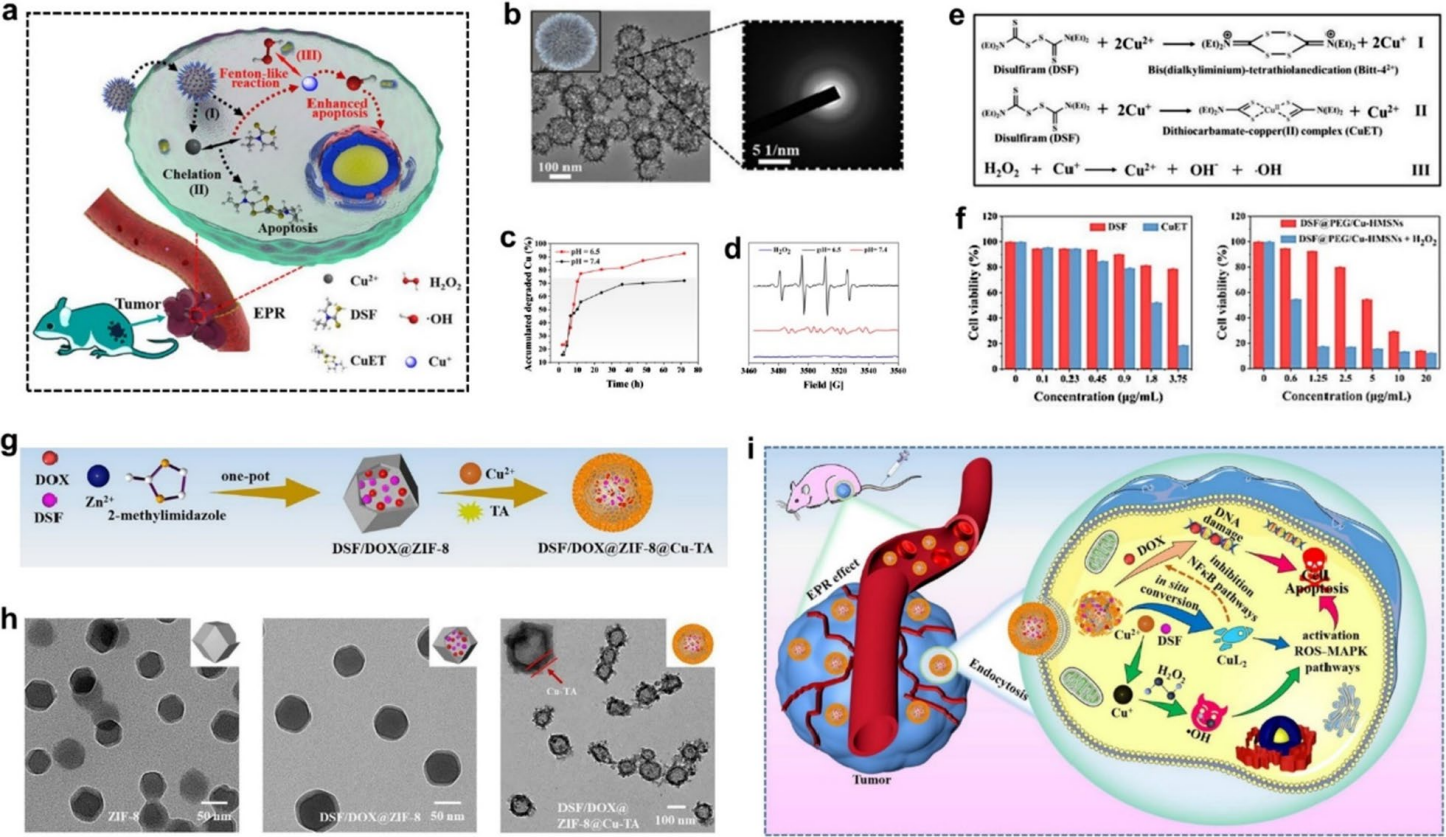
**a** Schematic diagram of the in vivo working mechanism of DSF@Cu-HMSNs enhanced chemotherapy. **b** TEM image and SAED pattern of PEG-Cu-HMSNs. **c** Accumulated release of copper ions from PEG-Cu-HMSNs at varied pHs. **d** The electron spin resonance (ESR) spectroscopy under different conditions (only H_2_O_2_; DSF@PEG/Cu-HMSNs + H_2_O_2_, pH = 7.4; DSF@PEG/Cu-HMSNs + H_2_O_2_, pH = 6.5). **e** Chemical mechanism of Cu-mediated reactions. **f** Cell viabilities of 4T1 cells after different treatments. **g** Schematic illustration of the synthesis of DSF/DOX@ZIF-8@Cu-TA. **h** TEM images of different nanoparticles. **i** Schematic diagram of the in vivo DSF/DOX@ZIF-8@Cu-TA triggered synergistic chemotherapy and CDT

In situ production of highly toxic CuET complexes and ROS inside tumor endows the nanosystem with both CDT-amplified chemotherapeutic effects and the biosafety for tumor-specific therapy (Fig. 4a). The sea urchin-like Cu-PEG/HMSNs (Fig. 4b) possess low crystallinity engineered framework that facilitates the rapid degradation of nanocarriers in an acidic TME (Fig. 4b, c). The chelation between the co-released DSF and Cu^2+^ enables the generation of therapeutic CuET. Interestingly, the Cu^2+^ is concurrently reduced into Cu^+^ ions by DSF chelation that can further trigger Fenton-like reactions to produce high cytotoxic •OH from intratumoral overexpressed H_2_O_2_ (Fig. 4d, e). The in vitro cytotoxicity assay further demonstrated that the anti-cancer effect of DSF@PEG/Cu-HMSNs was greatly reinforced after adding H_2_O_2_, manifesting the significant synergistic effect of CDT and DSF-mediated chemotherapy on tumor-specific treatment (Fig. 4f). Recently, Wang et al. prepared a novel pH-responsive metal–organic framework nanoparticle (denoted as DSF/DOX@ZIF-8@Cu-TA) to realize CDT-augmented chemotherapy [34]. DSF/DOX@ZIF-8 was fabricated by encapsulating DSF and doxorubicin (DOX) into zeolitic imidazole framework-8 (ZIF-8) via the one-pot method, followed decorated by copper ionic Cu^2+^-tannic acid (TA) (Fig. 4g). After being coated with Cu-TA composite material, the size of DSF/DOX@ZIF-8 NPs increased from 86.5 to 99.2 nm, accompanied by a morphological change from a hexagonal structure to a uniform core–shell spherical structure (Fig. 4h). With the assistance of ZIF-8 NPs mediated EPR effect, encapsulated DSF and DOX could be efficiently delivered to the tumor site. In mildly acidic TME, DSF/DOX@ZIF-8@Cu-TA continuously releases Cu^2+^, DSF, and DOX in situ, triggering the intratumoral DSF-Cu^2+^ chelating to produce highly toxic CuL_2_ and ROS (Fig. 4i). More importantly, the ROS-MAPK and NF-κB signaling pathways can be also inhibited by CuL_2_, which could further boost the anti-cancer effect of DOX, thereby significantly augmenting the therapeutic efficiency of CDT-coordinated chemotherapy. In the meantime, the prepared nanoparticles did not induce significant in vivo toxicity. This “non-toxicity to toxicity” transformation triggered by in situ chelation reactions presents a distinctive paradigm in tumor treatment strategies and offers the opportunity to promote CDT-based combination chemotherapy.

### Nanomedicine-mediated synergistic chemotherapy and PTT

PTT has received widespread attention in tumor treatment as one of the physiotherapeutic tools for tumors, by using the near-infrared (NIR) laser to convert light energy into heat, which can rapidly eliminate tumor cells and partially overcome tumor recurrence in a short period of time. Meanwhile, the locally generated heat can act as a stimulus to accelerate the responsive release of loaded drugs as well as the in situ conversion of the nanomedicine. Thus, the combination of PTT and.

chemotherapy can give full play to their respective advantages and overcome disadvantages, greatly improving the anti-tumor effect. [35, 36].

**Fig. 5:**
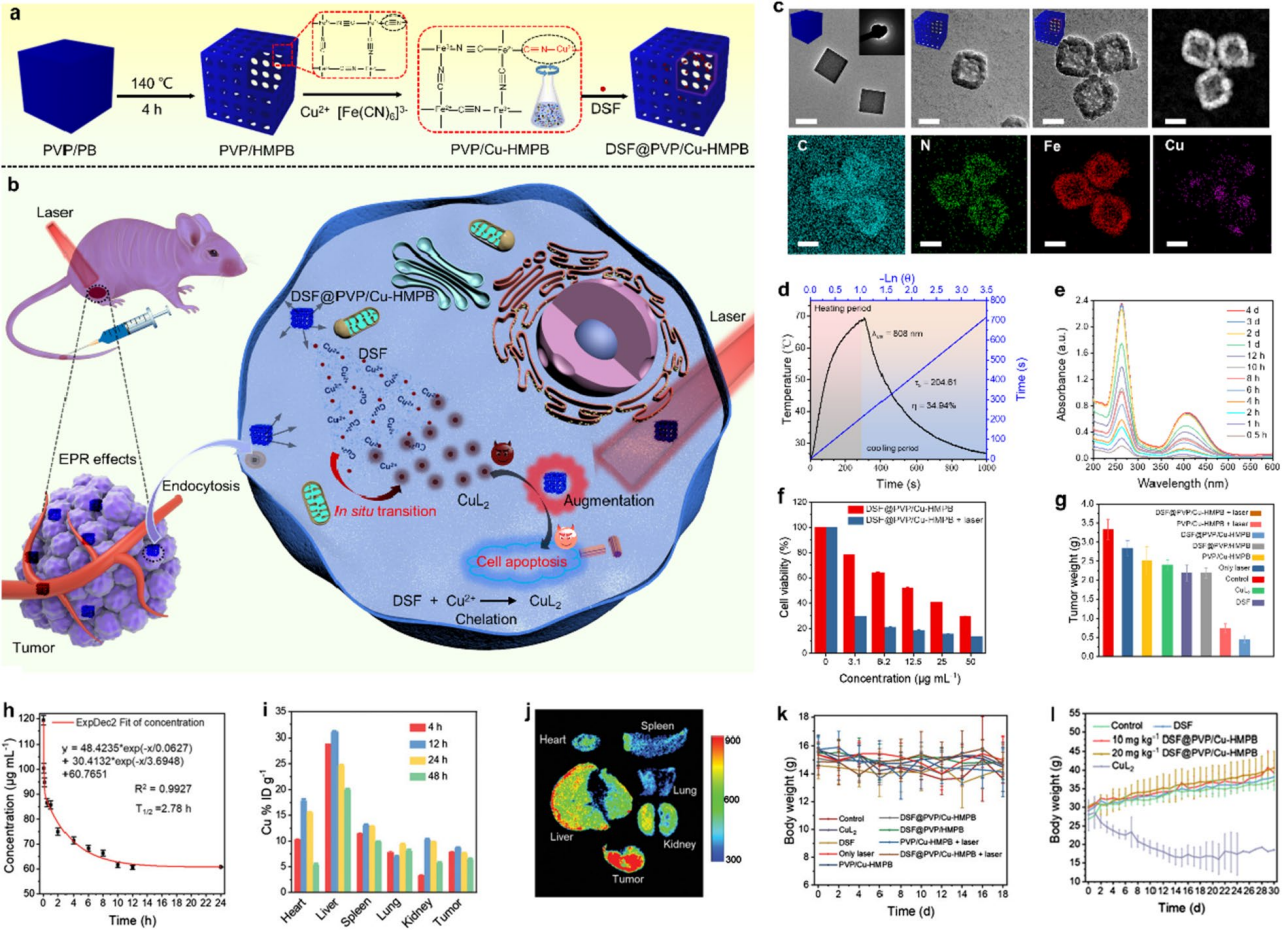
Cu-engineered HMPB for PTT enhanced intratumoral chelating for tumor-specific synergistic therapy based on the nontoxicity-to-toxicity transition of DSF. **a** Schematic diagram of the fabrication of DSF@PVP/Cu-HMPB and **b** the in vivo PVP/Cu-HMPB triggered PTT enhanced chemotherapy. **c** TEM images and corresponding elemental mapping of DSF@PVP/Cu-HMPB. Scale bars 50 nm. **d** Temperature change curves of PVP/Cu-HMPB under light irradiation and subsequently after turning off the light source. **e** Absorbance of DSF@PVP/Cu-HMPB solution in the range of 200–600 nm at different time intervals. **f** Cell viabilities of 4T1 cells after being treated by different conditions for 24 h. **g** Weight of tumors in different groups of BALB/c nude mice at the end of treatment. **h** The blood-circulation lifetime of PVP/Cu-HMPB. **i** Biodistribution of PVP/Cu-HMPB-derived Cu^2+^ at different time durations after injecting PVP/Cu-HMPB. **j** Bioluminescence images of mice's main organs. **k**, **l** Body weight of mice in different groups

Recently, various novel nanomedicines have been developed to optimize anti-cancer efficacy through the synergistic effects of PTT and chemotherapy. Most recently, great efforts have been made to develop novel nanomedicines in order to boost the anti-tumor outcomes through the synergistic effect between PTT and chemotherapy, and significant achievements have been obtained. Recently we have designed a copper dopped hollow mesoporous Prussian blue (Cu-HMPB)-based nanomedicine for in situ chemical reaction-activated and hyperthermia-enhanced chemotherapy of the above-mentioned DSF [37]. In this research, the Cu-HMPB was designed and constructed to load DSF forming DSF@PVP/Cu-HMPB for photothermal augmenting DSF chemotherapy via DSF-Cu^2+^ chelating reaction (Fig. 5a). After accumulating at tumor regions, the mild acidic TME-sensitive Cu^2+^-doped DSF@PVP/Cu-HMPB framework rapidly collapsed, accelerating the release of DSF while ensuring the adequate supply of Cu^2+^ within the tumor. Resultantly, the released DSF was turned into CuL_2_ in-situ by chelating co-released Cu^2+^ for killing tumor cells. Notably and importantly, on the basis of its inherent photothermal-conversion effect, the PVP/Cu-HMPBs carriers significantly amplified the chemotherapeutic efficacy of DSF-derived CuL_2_ under NIR irradiation (Fig. 5b). TEM images show that these synthesized PVP/Cu-HMPB nanoparticles feature a hollow mesoporous cuboidal nanostructure which is favorable for drug loading, while dark-field image and corresponding energy-dispersive X-ray spectroscopy (EDS) mappings further demonstrate the success of Cu^2+^ doping in its frameworks (Fig. 5c). After the doping, the ability to convert light energy into the heat of PVP/HMPB was not destroyed, and the photothermal-conversion efficiency was calculated to be as high as 34.94%, enabling DSF@PVP/HMPB to efficiently and continuously generate abundant heat under NIR irradiation (Fig. 5d). After DSF@PVP/Cu-HMPB was immersed in weakly acidic SBF at pH 6.0, the characteristic absorption peaks of CuL_2_ were intensified with the extension of the incubation time interval, demonstrating the cumulative formation of CuL_2_ (Fig. 5e). These results also demonstrate that the conceived therapeutic strategy of in situ transition of DSF into highly toxic CuL_2_ triggered by PVP/Cu-HMPB nanomedicine is feasible in vitro. More interestingly, DSF@PVP/Cu-HMPB achieved remarkably enhanced anticancer effects in killing tumor cells in vitro and inhibiting tumor growth in vivo upon NIR irradiation, (Fig. 5f, g). The long blood-circulation half-time (2.87 h) of the carrier PVP/Cu-HMPB provided a long enough time frame for the effective accumulation of DSF@PVP/Cu-HMPB inside the tumor, and the passive-targeting efficiency of DSF@PVP/Cu-HMPB reached 8.65% in 12 h post-injection (Fig. 5h, i). The in vivo fluorescence imaging (Fig. 5j) also validated the efficient accumulation of DSF@PVP/Cu-HMPB within the tumor. Upon laser irradiation, DSF@PVP/Cu-HMPB achieved remarkably enhanced anticancer effects via in situ generating highly toxic CuL_2_. Importantly, the body weights of the mice in all groups showed no significant changes, indicating negligible systemic toxicity of the preprepared nanoparticles (Fig. 5k). The quantitative comparisons of the designed nanoparticles versus conventional chemotherapeutic in organ accumulation and in vivo toxicity were also assessed by injecting DSF@PVP/Cu-HMPB and CuL_2_ into healthy mice, where CuL_2_ showed significant in vivo toxicity (Fig. 5l). In a follow-up study, were Liu et al. designed and prepared DSF-loaded hollow copper sulfide nanoparticles (DSF@PEG-HCuSNPs) to achieve photonic hyperthermia-amplified cancer chemotherapy of DSF, further validating the remarkable synergetic effects between the chemical reactions-triggered DSF chemotherapy and PTT. [38].

**Fig. 6:**
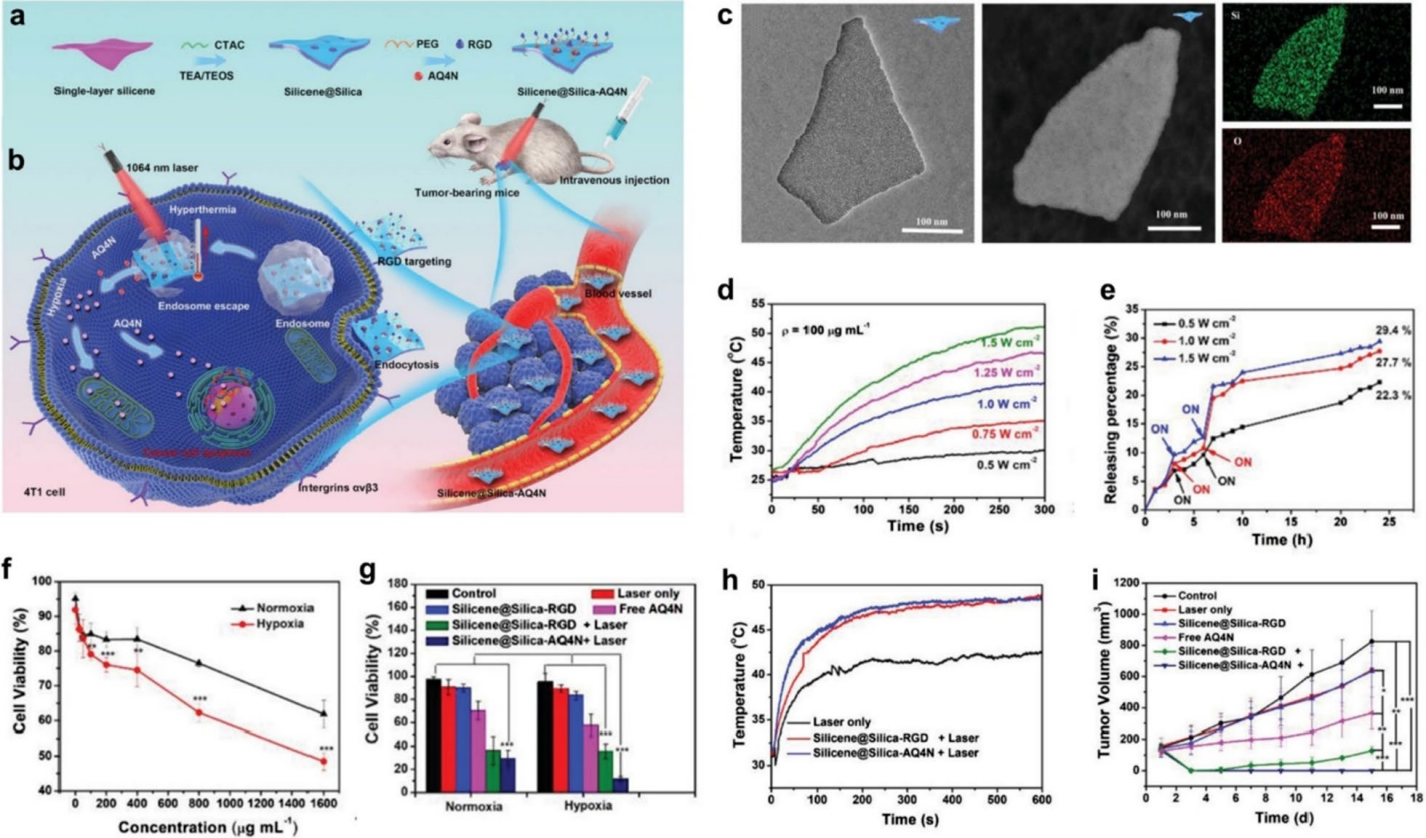
Design principle and therapeutic mechanism of Silicene@Silica-AQ4N. **a** Synthetic route of Silicene@Silica-AQ4N. **b** Illustration of the Silicene@Silica-AQ4N triggered synergistic photonic hyperthermia amplified chemotherapy in vivo. **c** The TEM, Dark-field TEM images, and corresponding elemental mapping of Silicene@Silica nanosheets. **d** Temperature change curves of Silicene@Silica-RGD under laser irradiation with different power densities. **e** The drug release of Silicene@Silica-AQ4N in PBS (pH = 7.4) under irradiated with laser irradiation with different power densities. **f**, **g** Cell viabilities of 4T1 cells treated by different conditions. **h** Temperature change curves at the tumor site of mice during different treatments. **i** Tumor volume of mice in different groups

The strategy for tumor-specific delivery and controlled release of prodrugs, and further in situ activation of the prodrugs is also an effective means of reducing the toxic side effects of conventional chemotherapy [39]. Light featuring flexible tenability, noninvasiveness, and site-specific controllability can be used as a convenient switch to control the precise release of chemotherapeutic agents [40]. In this context, Xu et al. fabricated a NIR-responsive nanomedicine based on the 2D mesoporous core/shell-structured multifunctional theranostic nano-platform (Silicene@Silica) to achieve NIR laser-controlled prodrug banoxantrone dihydrochloride (AQ4N) release and combined thermo-chemotherapy (Fig. 6a) [41]. After accumulation in the tumor, the “core” (silicene) of 2D core/shell-structured nano-platform with RGD peptide-targeted modification could rapidly achieve photothermal transformation upon NIR-II laser irradiation, which further exacerbates hypoxia in TME by disrupting tumor microcirculation, and then further effectively activates the hypoxia-sensitive drug AQ4N in the mesoporous silica "shell" for efficient hypoxia activation and PTT-enhanced chemotherapy. (Fig. 6b). As Fig. 6c displays, the single-layered.

silicene nanosheets coated with a mesoporous silica layer were successfully synthesized, where the mesoporous silica layer provides desirable reservoirs for the loading of AQ4N. As a novel photothermal agent, the 2D silicene “core” of the constructed nanoplatform could rapidly produce heat shock upon NIR-II laser irradiation in vitro, which would trigger the pH-responsive release of AQ4N (Fig. 6d), facilitating the high tumor-specific chemotherapy (Fig. 6e). The results of cellular experiments showed that the anti-cancer effect of AQ4N was greatly enhanced under hypoxic conditions (Fig. 6f). Especially, the anticancer efficacy of Silicene@Silica-AQ4N could be further augmented after NIR irradiation under hypoxic conditions (Fig. 6g), manifesting the significant synergetic cancer-killing effects in vitro. After the nanosheets are enriched into the tumor site through the RGD-mediated active-targeting effect, Silicene@Silica-AQ4N generates abundant thermal under NIR irradiation to trigger the rapid release of prodrug AQ4N from the mesoporous silica layer and concurrently amplify the hypoxia of TME by destroying the tumor microcirculation (Fig. 6h). The PTT-intensified hypoxic environment in tumor tissue effectively activates AQ4N for effectively killing residual tumors from PTT and preventing tumor recurrence (Fig. 6i).

### Nanomedicine-mediated synergistic chemotherapy and PDT

Non-invasive photodynamic therapy (PDT), by leveraging light to activate photosensitizers (PS) to produce cytotoxic ROS that causes tumor cell death via the peroxidation of lipids, protein, DNA, and RNA, has been considered an attractive strategy for clinical cancer therapy [42]. On the other hand, the produced ROS can act as the specific stimulus to initiate the controlled release of drug payloads by disassembling the covalent bioconjugation of chemotherapeutics and photosensitizers, thus laying the foundation for synergistic chemotherapy and PDT [43–45]. Accumulating clinical data, supported by the results of pre-clinical studies in animals, suggest that PDT can rationally cooperate with standard chemotherapy to achieve better results.

**Fig. 7:**
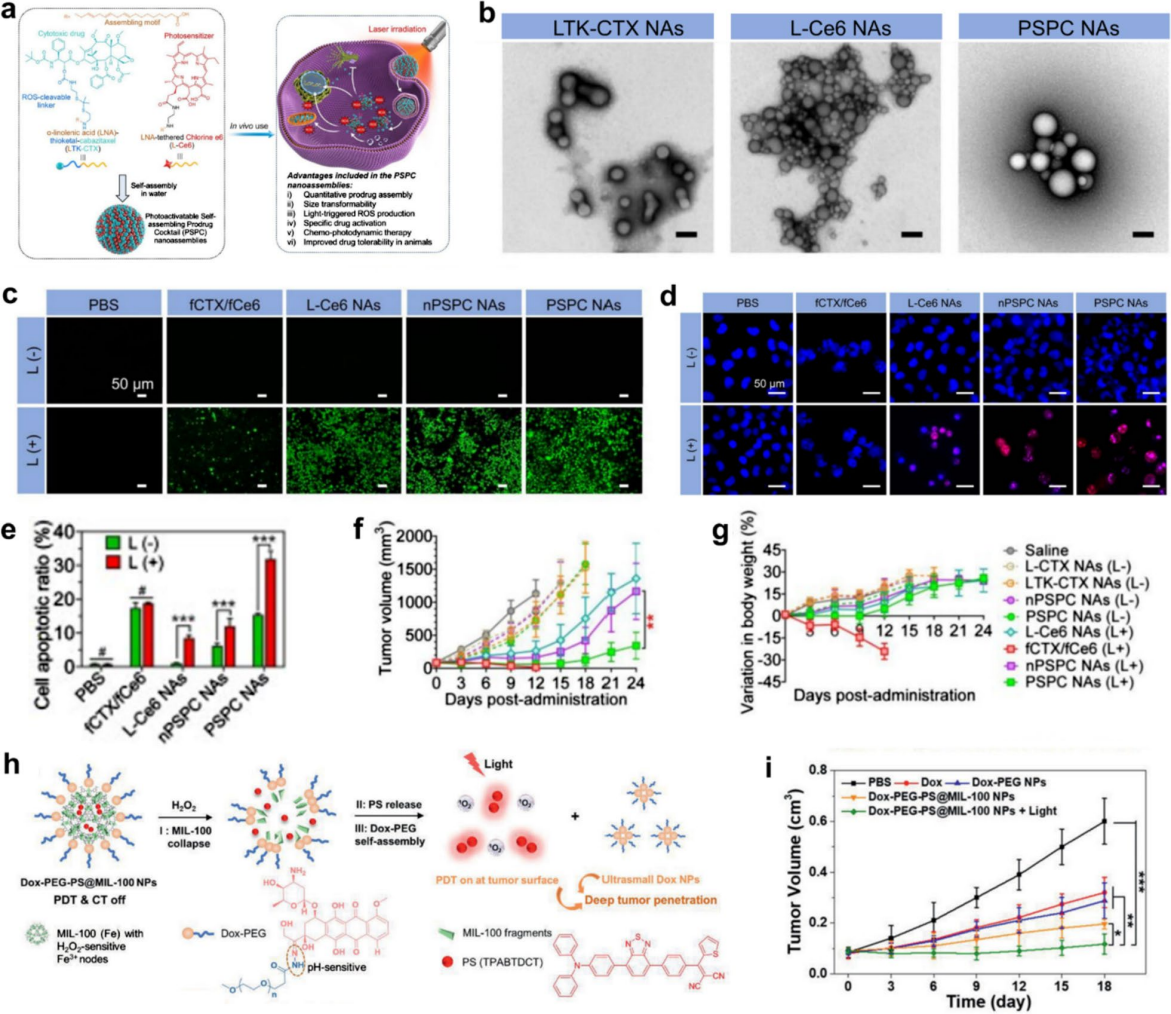
**a** Schematic representation of molecular structures of PUFAylated cabazitaxel prodrug (LTK-CTX) and chlorin e6 prodrug (L-Ce6) and the working mechanism of PSPC nanoassemblies in vivo. **b** Morphology presentation of the LTK-CTX nanoassemblies (NAs), L-Ce6 NAs, and PSPC NAs. Scale bars, 100 nm. **c** Confocal imaging of A375 cells stained with DCFH-DA for 30 min after different treatments. **d** Confocal imaging of A375 cells stained with γ-H2AX (red) and DAPI(blue) in different groups. **e** Flow cytometry analysis of A375 cell apoptosis treated by varied conditions. **f** Tumor volume change curves of mice in different groups. **g** Body weight change of mice in different groups post-administration. **h** Scheme showing different stages of Dox-PEG-PS@MIL-100 NPs in H_2_O_2_ and corresponding size change and PDT assisted tumor penetration. **i** The tumor volumes change curves of mice

Recently, Huang et al. prepared a photoactivatable self-assembling prodrug cocktail (PSPC) nanoassemblies for synergistic tumor therapy [46]. The PSPC nanoassemblies were formed by the self-assembly of the two assembling motives of α-linolenic acid (LNA)-thioketal-cabazitaxel (LTK-CTX) prodrug and LNA-conjugated photosensitizer chlorine e6 (termed L-Ce6). After PSPC nanocomponents were taken up by tumor cells, the co-assembled L-Ce6 immediately generated abundant ROS under NIR exposure that was able to not only activate the cytotoxic of cabazitaxel through the rapid cracking of the TK bonds but also synergize with PDT for high-efficiency tumor therapy (Fig. 7a). TEM-based morphological investigations (Fig. 7b) verify that both LTK-CTX and L-Ce6 monomer conjugates are capable of forming spherical nanostructures by self-assembly in water. Moreover, L-Ce6 conjugate is miscible with the LTK-CTX conjugate at arbitrary molar ratios to obtain PSPC nanoassemblies. The three carboxyl groups of free Ce6 (fCe6) prevent them from being absorbed by.

negatively charged cancer cells, thus resulting in undesirable ROS generation upon laser irradiation. In contrast, L-Ce6 self-assembled nanoparticles can be rapidly uptaken by cancer cells through the endocytosis pathway to exert effective PDT. As shown in intracellular ROS detection experiments using DCFH-DA as an indicator of ROS green fluorescence, L-Ce6 nanoassemblies pretreated cells yield a strong green fluorescence upon laser irradiation, demonstrating that L-Ce6 assemblies can indeed generate substantially higher concentrations of ROS in cells than fCe6 due to the increased cellular-uptake (Fig. 7c). In combination with ROS-activated CTX, PSPC NAs can cause maximum oxidative damage to DNA and ultimately lead to tumor cell apoptosis under laser excitation (Fig. 7d, e). In vivo experiments further demonstrated the significant synergistic therapeutic effect of PSPC NAs-mediated PDT and chemotherapy without significant toxicity issues compared to free drug combination therapy that induced weight loss and death in mice (Fig. 7f, g). In addition, Wang et al. reported Dox-PEG-PS@MIL-100 nanoparticles (NPs) based nanocatalytic medicine for effective photo-chemotherapy in the deep tumor [47]. This elaborate nanomedicine was formed by using pH-sensitive Dox-PEG to encapsulate the photosensitizer (PS)-loaded MIL-100 NPs. As Fig. 7h displayed, Dox-PEG-PS@MIL-100 NPs can degrade into fragments in response to intratumoral H_2_O_2_ and specifically release PSs at the tumor surface for effectively generating ROS upon laser irradiation. In the meantime, exterior Dox-PEG is able to self-assemble into ultrasmall Dox NPs after the collapse of PS@MIL-100 that can be beneficial for deep tumor penetration and chemotherapy. Dox-PEG-PS@MIL-100 NPs have been demonstrated to be capable of realizing effective photo-chemotherapy in deep tumors with negligible side effects by detailed in vivo experiments (Fig. 7i).

### Nanomedicine-mediated synergistic chemotherapy and starvation therapy

**Fig. 8:**
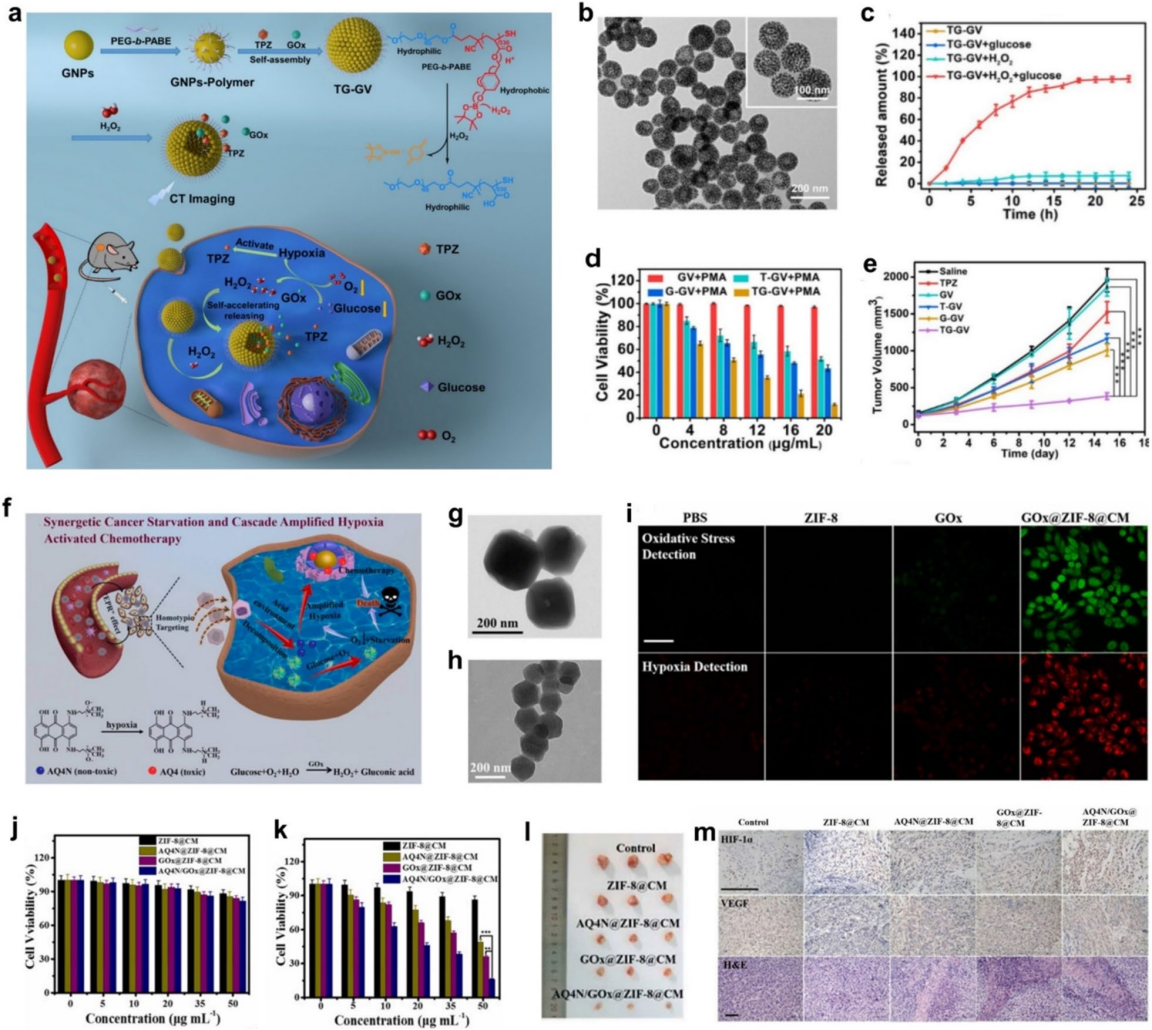
Nanomedicine-enabled tumor synergistic starvation/chemotherapy based on Gox-mediated glucose glycolysis. **a** Schematic diagram of the synthesis and use of TG-GV for combination chemo/starvation therapy. **b** TEM image of TG-GVs. **c** The release curves of TPZ from TG-GVs solutions with or no glucose addition. **d** The cell viabilities of 4T1 cells after undergoing various treatments for 24 h. **e** The recorded tumor volume change in different groups during the treatments. **f** Schematic of in vivo working mechanism of A/Q4N/GOx@ZIF-8@CM. **g**, **h** TEM images of AQ4N/GOx@ZIF-8 and AQ4N/GOx@ZIF-8@CM. **i** CLSM images of HepG2 cells stained with oxidative stress/hypoxia detection probe after treated by different conditions. Scale bar, 20 μm. **j**, **k** Viabilities of LO2 and HepG2 cells after various treatments. **l** The photographs of tumors tissues. **m** HIF-1α, VEGF immunohistochemical, and H&E staining images of tumor tissues

Based on the fact that the Warburg effect of tumors is caused by inefficient aerobic glycolysis making tumor cells more dependent on glucose than normal tissue cells, glucose oxidase (GOx)-mediated starvation therapy has been demonstrated to be an efficient tumor therapy modality by elevating intratumoral glucose consumption [48–50]. In particular, the GOx-mediated glycolysis can be employed to alter the TME by raising the concentration of H_2_O_2_ and enhancing intracellular hypoxia, which can be exquisitely used to control the release and activation of therapeutic agents for effective combination therapy [51]. Nanomedicine-based multimodal cancer starvation strategies to enable synergistic cancer therapy have been developed, especially for starvation-synergized chemotherapy, which has been demonstrated to be an efficient way to minimize the side effects of free chemodrugs and result in superadditive therapeutic.

effects. According to this, Tang et al. presented the self-accelerating H_2_O_2_-responsive plasmonic nanovesicles (TG-GVs) for synergistic chemo/starving therapy of tumors [50]. The GOx enzymes and tirapazamine (TPZ) prodrug were simultaneously encapsulated in the plasmonic gold nanovesicles (GVs) during the assembly. The TG-GVs showed excellent stability and no noticeable leakage of the encapsulated molecules for days. The overexpressed H_2_O_2_ within tumor triggered the GOx release from the TG-GVs, which then catalyzed the oxidation of glucose by oxygen to amplify intratumoral hypoxia that subsequently activates the hypoxia-sensitive prodrug TPZ and then to generate highly toxic free radicals for chemotherapy. The H_2_O_2_ generated in the redox reaction further accelerates the dissociation of vesicles and facilitate the co-release of GOx and TPZ, thus continually amplifying the starvation/chemotherapy combined antitumor efficacy (Fig. 8a). TEM image shows that the GVs possess a hollow cavity which is beneficial for cargo loading (Fig. 8b). In vitro payload release results show that H_2_O_2_ produced in the glucose oxidation reaction has triggered the release of 84% of the loaded TPZ from the TG-GVs (Fig. 8c). Meanwhile, cellular and in vivo experiments further demonstrate the superadditive anticancer efficacy between GOx mediated starvation therapy and tumor-specific TPZ chemotherapy (Fig. 8d, e). In the latest research, Shao et al. reported a biomimetic nanoreactor (AQ4N/GOx@ZIF-8@CM) based on GOx and banoxantrone (AQ4N) encapsulated metal–organic framework ZIF-8 which was further modified by the cancer cell membrane (CM) for starvation therapy synergized and cascade amplified hypoxia-activated chemotherapy [52]. The encapsulated Gox and AQ4N in ZIF-8 will not release until they reach the tumor site, for ensuring the tumor-specific synergistic starving/chemotherapy within tumoral mildly acidic microenvironment (Fig. 8f). Obvious wrinkles can be observed on the edge of AQ4N/GOx@ZIF-8@CM, indicating that CM has been successfully coated on the uniform polyhedron morphology AQ4N/GOx@ZIF-8 nanoparticles (Fig. 8g, h). These CLSM images show that the loaded GOx not only enables tumor cells to starve but also elevates intracellular oxidative stress/hypoxia levels to trigger the resulting hypoxia-activated chemotherapy (Fig. 8i). Due to the relatively low accumulation of nano-reactors in LO2 cells, AQ4N/GOx@ZIF-8@CM can selectively kill tumor cells without significant cytotoxicity to normal cells, indicating the excellent biocompatibility of the prepared material (Fig. 8j, k). Importantly, the in vivo treatment experiments showed that AQ4N/GOx@ZIF-8@CM significantly inhibited tumor growth by triggering tumor-specific synergistic starvation therapy and cascade amplification of hypoxia-activated chemotherapy, (Fig. 8l, m).

**Fig. 9:**
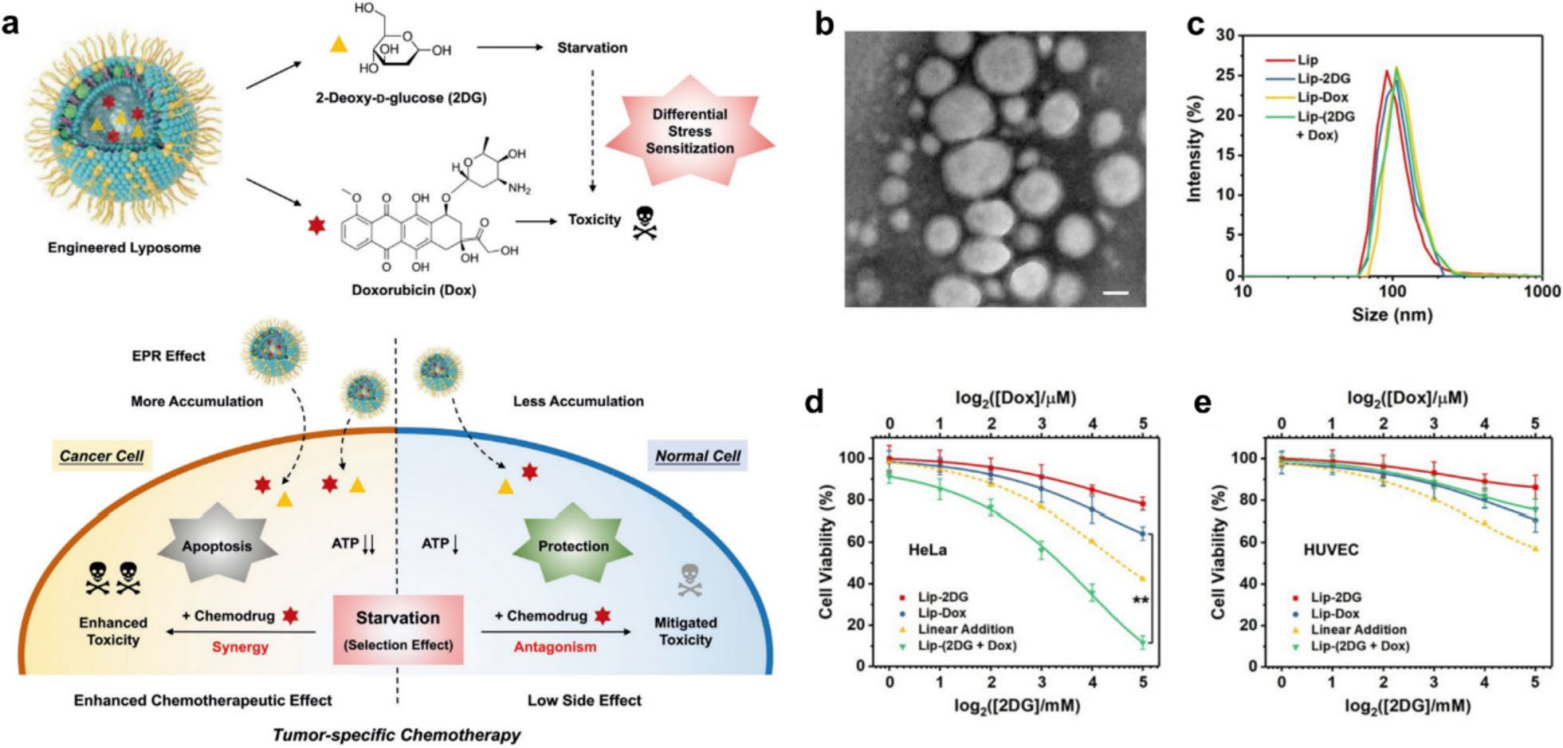
Nanomedicine induced starvation to generate differential stress sensitization for enhanced tumor-specific chemotherapy. **a** Schematic representation of the fabrication of liposomal nanomedicine and its interactions with tumor or normal cells. **b** TEM -based study of Lip-(2DG + Dox). Scale bar, 50 nm. **c** DLS measurements of different liposomes. **d**, **e** Cell viabilities of HeLa tumor cells and normal HUVEC cells after different treatments

Previous studies have shown that fasting can significantly inhibit tumor growth and sensitize various types of neoplastic cells to chemotherapy drugs while protecting normal cells [53, 54]. Intrinsic genomic differences between cancer and normal cells contribute to this differential stress sensitization to chemotherapy, wherein active oncogenes in cancer cells in the presence of nutritional deficiency will promote a redistribution of energy from the maintenance pathway to the growth pathway, resulting in diminished chemoresistance. Conversely, the nutritional deficiency will cause normal cells switch to a self-protective mode. Although fasting combined with chemotherapy for cancer treatment is already in clinical trials, fasting is still less toleratable by patients due to severe food deprivation. Hence, non-dietary tumor-specific starvation therapy based on nanomedicines would be more conducive to improving anti-cancer efficacy while mitigating side effects. With the aid of 2DG to induce starvation and generate differential stress sensitivity between cancer and normal cells, our group constructed a 2-deoxyglucose (2DG) and doxorubicin (Dox) co-loaded liposome nanosystem (called Lip-(2DG + Dox)) to achieve differential stress-sensitive tumor-specific chemotherapy (Fig. 9a) [55]. These quasi-spherical nanoparticles of less than 200 nm in diameter can be effectively internalized by cancer cells in vitro and thus passively accumulate in the tumor by EPR effect in vivo (Fig. 9b, c). Upon entry into tumor cells, 2DG in Lip-(2DG + Dox) acts as a glucose analog that is capable of blocking the generation of adenosine 5'-triphosphate (ATP) in glycolysis, thereby effectively predisposing cancer cells to short-term severe starvation and inducing subsequent differential stress sensitization process. For tumor cells, however, the cytocidal effect of Lip-(2DG + Dox) is much more pronounced than the linear additions of Lip-Dox and Lip-2DG alone, further confirming the therapeutic synergy between the glycolytic inhibition of 2DG and the cytotoxicity of Dox, demonstrating a significant superadditive (“1 + 1 > 2”) anti-tumor effect (Fig. 9d). Of note, the normal cell line shows largely higher viability after 24 h incubation with Lip-(2DG + Dox), suggesting that the starvation protection mechanism triggered by 2DG has significantly reduced the toxicity of Dox in normal cells (Fig. 9e). Such a differential stress-sensitization strategy offers a perspective approach to augment cancer therapy by selectively protecting normal cells rather than enhancing the toxicity of conventional chemical drugs against malignant cells, providing a promising paradigm for the design of future nanomedicines of enhanced bio-safety.

### Nanomedicine-mediated synergistic chemotherapy and immunotherapy

Cancer immunotherapy, which fights against cancer by harnessing the power of the host immune system, is a revolutionary advance in cancer research, yet is often undercut by low patient response rates or potential immune-related adverse events in clinical treatment. [56, 57] Chemotherapy has been proven in recent years to be able to trigger immunogenic cell death (ICD) of tumor cells, a unique cell death pathway associated with endogenous danger signals released by dying cells that boost the anti-cancer immunity of immunocompetent hosts [58]. The robust tumoral ICD will stimulate the exposure of more tumor-associated antigens (TAAs) by expressing relevant protein markers, thus contributing to the maturation of dendritic cells (DCs) and subsequent presentation of TAAs from mature DCs to T cells. Concurrently, pro-inflammatory cytokines released from dying tumor cells can repolarize pro-tumorigenic M2 macrophages to anti-tumorigenic M1 phenotype, evoking innate immunity. In this regard, the activation of ICD-triggered powerful anticancer immunity is a highly promising and viable therapeutic strategy to synergistically amplify the tumor inhibition effects of nanomedicine.

**Fig. 10:**
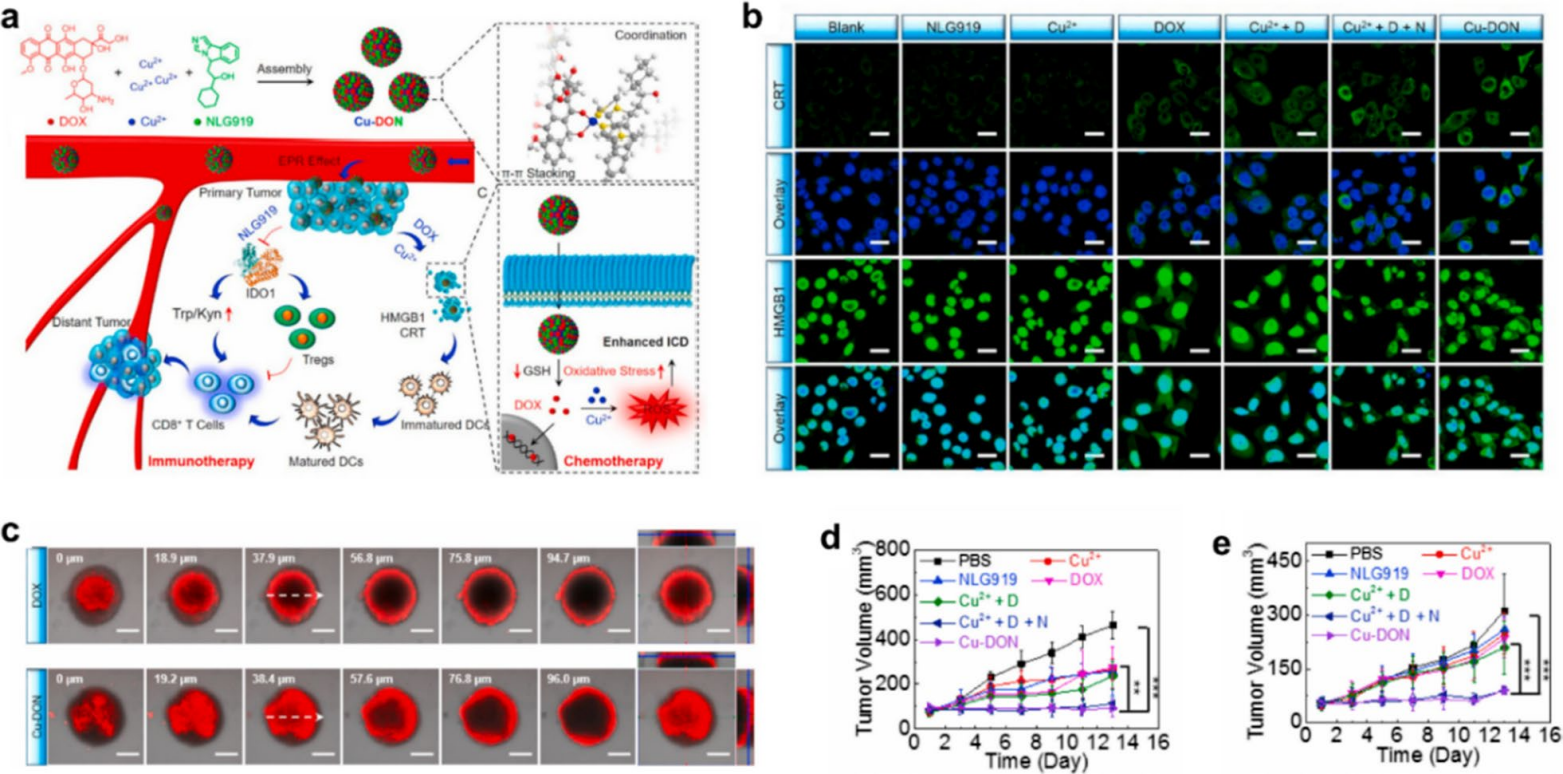
A novel nanomedicine based on the self-assembling among Cu^2+^, DOX, and NLG919 for chemotherapy sensitized immunotherapy. **a** Schematic diagram of the synthetic route and in vivo working mechanism of Cu-DON. **b** Immunofluorescence images of CRT and HMGB1 in 4T1 cells after different treatments. Scale bar, 25 μm. **c** Z-stack CLSM images of 4T1 tumor spheroids after being treated by DOX and Cu-DON. Scale bar, 200 μm. **d**, **e** Tumor volume change curves of the primary tumor and distant tumor volume

Among the broad spectrum of chemotherapeutic drugs, doxorubicin (Dox) has been proven capable of inducing ICD, thereby eliciting certain levels of antitumor immune responses accompanying chemotherapy [59–61]. By virtue of the π-π stacking and coordination effect among Cu^2+^, DOX and NLG919, Zhao et al. designed and prepared a self-assembled nanosized oxidative stress amplifier (designated as Cu-DON) to achieve chemotherapy sensitized immunotherapy [62]. Following accumulation of Cu-DON NPs at tumor tissue via classical EPR effects, their GSH-responsive drug release behavior will initiate DOX-based immunogenic chemotherapy to inhibit primary tumor. Co-released Cu^2+^-mediated GSH depletion will further amplify DOX-induced oxidative stress, leading to enhanced ICD. Furthermore, concomitantly delivered NLG919 will inhibit indoleamine 2,3-dioxygenase 1 (IDO-1), resulting in immune response reactivation for fighting primary and distant tumors by remodeling the immunosuppressive tumor microenvironment (ITM). Immunofluorescence staining revealed that Cu-DON could induce a strong ICD response in tumor cells, as evidenced by effective calmodulin (CRT) exposure and high mobility group box 1 (HMGB1) release of 4T1 cells after Cu-DON treatment, which are pivotal biomarkers of ICD (Fig. 10b). Compared with free DOX, nanosized Cu-DON has a stronger tumor penetration, which facilitates deep tumor chemotherapy while evoking the powerful immune response in vivo (Fig. 10c). Overall, this overwhelmingly superior oxidative stress amplification strategy for chemosensitizing immune response provides new insights into the development of self-delivering nanoplatforms for precision oncology therapy (Fig. 10d, e).

**Fig. 11:**
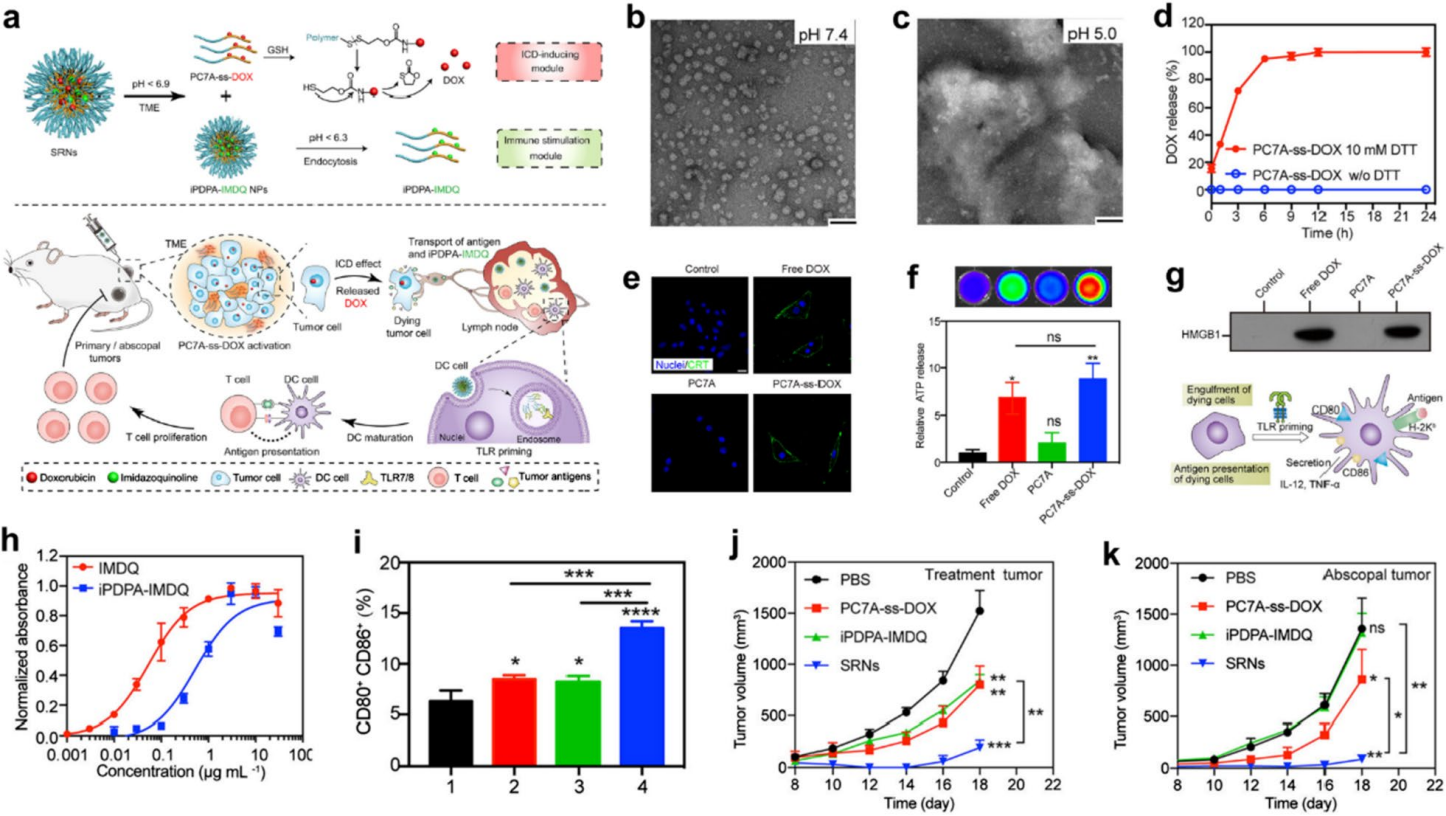
Engineered sequential pH/redox responsive nanoparticles (PC7A-ss-DOX) for precisely delivering chemodrug (DOX) and immunoadjuvant (imidazoquinolines, IMDQs) for synergistic chemo-immunotherapy. **a** Schematic diagram of the chemical structure of PC7A-ss-DOX and the sequential immune activation mechanism by PC7A-ss-DOX. **b**, **c** TEM images of PC7A-ss-DOX at different pH values. Scale bar, 100 nm. **d** In vitro DOX release from PC7A-ss-DOX under different conditions. **e** Immunofluorescence images of CRT after various treatments. Scale bar, 20 μm. **f** ATP levels in tumor cells after different treatments. **g** The content of HMGB1 in the tumor cells treated by different conditions and schematic diagram of DC maturation under TLR priming. **h** RAW-Blue reporter cells number after being stimulated by different materials. **i** Percentage of mature DCs in different groups: (1) B16-OVA; (2) PC7A-ss-DOX treated B16-OVA; (3) B16-OVA + iPDPA-IMDQ; (4) PC7A-ss-DOX treated B16-OVA + iPDPA-IMDQ. The tumor growth curves of **j** primary tumors upon different treatments and corresponding **k** distant tumors of mice

However, TME remodeling induced by ICD can only provide a sub-optimal outcome for cancer immunotherapy as a result of insufficient activation of DCs and T cells in lymph nodes [63]. An alternative cancer immunotherapy option of transferring antigens and pattern recognition receptor (PRR) agonists to DCs in lymph nodes via nanovaccine for facilitating antigen expression, DC maturation, T cell responses, and the extensive tumor recognition, has unfortunately failed due to high tumor heterogeneity within and between tumor types. It is therefore essential to develop an effective nanoparticle strategy to simultaneously achieve in situ release of tumor antigens and promote the maturation of antigen-presenting cells (APCs). Herein, Wang et al. synthesized pH/redox responsive nanoparticles (SNRs) to co-activate the antigen exposure of tumor cells and the maturation of dendritic cells (DCs) in lymph nodes by sequentially inducing activation of ICD and toll-like receptor 7/8 (TLR7/8) for synergistic chemo-immunotherapy (Fig. 11a) [64]. The prepared SRNs contain two functional modules of PC7A-ss-DOX and iPDPA-IMDQ, which are regarded as ICD-induction and immune stimulation module, respectively. Such self-assembled SRNs can remain stable in neutral solutions, but dissociate autonomously in slightly acidic environments and release the corresponding two functional modules (Fig. 11b, c). Subsequently, GSH overexpressed in tumors will further cleave the reduction-sensitive bond in the PC7A-ss-DOX module, releasing the chemotherapeutic drug DOX and inducing tumor ICD effect (Fig. 11d). Following the treatment with PC7A-ss-DOX, CRT exposure, as well as the release of adenosine triphosphate (ATP) and HMGB1 from B16-OVA cells, could be clearly detected, indicating that DOX released from PC7A-ss-DOX can effectively induce tumor ICD (Fig. 11e–g). Benefiting from conjugation to the carrier, iPDPA-IMDQ can activate Toll-like receptors (TLR7/8) of DCs in lymph nodes more effectively than free IMDQ 7/8, directly stimulating the maturation of DC cells (Fig. 11h). The combination between PC7A-ss-DOX pretreated dying tumor cells and iPDPA-IMDQ allows the harvesting of more mature DCs that present TAA to T cells in vivo (Fig. 11i). After the efficient immune system activation of mice by SRNs, the TAAs provided by dying primary tumor cells can be rapidly presented to T cells by mature DCs to promote the activation of cytotoxic T cells for inhibiting the development of abdominal tumors (Fig. 11j, k). This sequential response-based collaborative self-assembling nanoparticle strategy offers a potentially promising opportunity for nanomedicine to promote synergistic cancer chemoimmunotherapy.

## Conclusion and outlook

Despite the current emergence of various anti-cancer strategies, chemotherapy remains a powerful tool for killing malignant cancer cells. Through intensive research into the pathogenesis of cancer, a range of chemotherapeutic agents with different mechanisms of action have been successfully exploited, greatly enriching the chemotherapy options for specific types of tumors. While its importance cannot be overstated, chemotherapy appears to be a double-edged sword in the fight against cancer due to severe challenges such as insufficient delivery, unwanted side effects, and drug resistance [65]. There is an urgent need to surmount the predicament of highly efficient chemotherapy. Fortunately, however, the leap forward in developing multiple-functional nanomedicines has brought exciting promises for enhancing the efficacy of chemotherapy on malignant tumors while diminishing toxicities to healthy tissues [66]. As mentioned earlier, nanomedicine first promises to enable target delivery of drug molecules to solid tumors, which attribute to their unique nanosize that can improve the enrichment of therapeutic agents via EPR effect [2]. Strategies to convert non-/low-toxic intrinsic/delivered substances into effective therapeutic products through nanomedicine-triggered chemical reactions within the tumor have also been developed to further reduce the chemotoxicity associated with conventional chemotherapeutic agents. Meanwhile, single-mode therapy strategy often fails to obtain optimal clinical outcomes of cancer treatment, whereas the highly tunable physicochemical properties of nanomaterials make it easy to seamlessly integrate chemotherapy with other treatment modalities. In this review, we have systematically summarized the recent advances in nanomedicine-enabled chemotherapy and the synergistic cancer therapies based on such chemotherapies, including the combinations of chemotherapy with CDT, PTT, PDT, starvation therapy, and immunotherapy (Table 1). Although nanomedicines are inappropriately envisioned as a panacea initially, they actually cannot solve all the challenges in cancer chemotherapy, and as a matter of fact, the related researches are still in their infancy. Several critical issues remain to be addressed for accelerating the transition of nanomedicine-enabled chemotherapy from bench to bedside.

**Table 1:** Summary of typical chemotherapy-instructed synergized cancer therapy strategies

Therapy modality	Chemotherapeutic agents			Adjuvant components	Working mechanism	Refs
Single Chemotherapy	BTZ; DOX; CPT			PVCL-GMA NGs; RGD peptide; CaO_2_/MnO_2_;	Enhancing enrichment and penestration of drug in tumor	[22]; [23]; [24]; [28]
Chemotherapy-CDT	DSF; DOX			PVP/Cu-HMSNs; ZIF-8	The nontoxicity-to-toxicity transition of DSF and H_2_O_2_-responsive·OH generation	[33]; [34]
Chemotherapy-PTT	DSF; AQ4N			PVP/Cu-HMPBs; CuS Silicene@Silica	PTT-enhanced the release of chemotherapeutic agents and anti-cancer activity	[37]; [38]; [41]
Chemotherapy-PDT	Cabazitaxel; DOX			Ce6; TPABTDCT	PDT-activated the anti-cancer activity of prodrugs	[46]; [47]
Chemotherapy-Starvation therapy	TPZ; AQ4N; DOX			Gox; 2DG	Starvation-sensitized tumor cells to chemo-drugs	[50]; [52]; [55]
Chemotherapy- Immunotherapy	DOX			NLG919; IMDQ	Chemotherapy-induced robust tumoral ICD to activate anti-cancer immunity	[62]; [64]

1. The effective preparation strategies of nanomedicines are the key to making them promising in oncology chemotherapy. In this text, therapeutic agents are mainly loaded into nanocarriers through weak interactions such as physisorption or spatially restricted domains between the guest and hosts. In such weak interactions, the cargoes in the carrier may leak out before they reach the target site in vivo, causing undesirable side effects. Although researchers have synthesized and developed various organic nanoparticles via facile fabrication methods to encapsulate chemotherapeutic agents, the commonly used organic solvents or elevated temperatures during their construction process may deteriorate the biological activities of the loaded therapeutic agents, such as enzymes, small-molecule drugs, and antibodies [67]. Nanoparticle carriers and therapeutic agents combined together by strong chemical bonding have better metabolic kinetics, however, in this case, their specific release becomes less easy to achieve. Therefore, it is necessary to find more feasible synthetic and construction strategies to balance the stability and the active release of therapeutic agents in order to facilitate their further clinical translation.

2. Effective accumulation of nanomedicines is the premise for nanomedicine-enabled chemotherapy to obtain enhanced therapeutic outcomes [68]. However, most of the available nanomedicines accumulate in tumor tissue through EPR effect, where the accumulative efficiency of tumor-targeted accumulation is rather low (less than 10% via i.v. administration), hindering their further clinical application [69]. In addition, the EPR effect depends on the mature tumor microenvironment, which does not always occur in all tumor types, further blocking the nanomedicine-mediated chemotherapy. Alternatively, another active tumor-targeting effect of nanoparticles, namely "nanomaterial-induced endothelial leakage" (NanoEL), may need to be investigated in more depth to substantially elevate the tumoral accumulation efficiency of nanomedicines for enhanced therapeutic efficacy.

3. The conversion of intrinsic/delivered substance from non-/less toxic to highly toxic after endocytosed into the tumor tissue usually depends on metal ion-mediated chemocatalytic reactions [32]. To achieve this goal, a number of transition metal ions (Fe, Cu, Mn, etc.) with catalytic properties can be introduced into the nanomedicine system. The powerful catalytic activity of transition metal ions endows the transition metal-engineered nanomedicines a wide spectrum of redox properties that can catalyze specific reactions, especially the intratumoral redox reactions to generate ROS or other toxic substances in situ. Although these transition metal ions in nanomedicine can be released in a concentrated manner in the acidic tumor microenvironment, the transition metals, especially copper ions, may cause some unexpected damages to the body since these nanomedicines, sometimes difficult to degrade, will heavily accumulate in liver and spleen after administration [70–72]. Therefore, the clinical application of such nanomedicines remains challenging due to the potential metal toxicity during the prolonged exposure in vivo. In the future, with more advances in coordination chemistry, much more safe nanomedicines can be designed or invented to achieve chemotherapy-based synergistic cancer therapy with enhanced bio-safety, by rationally selecting the types of transition metals and organic ligands.

4. Nanomedicine-enabled chemotherapy in combination with other therapeutic modalities can achieve augmented therapeutic efficacy for cancer by different synergistic mechanisms. However, it is often difficult to find the most appropriate doses of multifunctional nanomedicines for achieving the optimized synergistic efficiency between the different treatment modalities in practical oncology therapeutic applications, resulting in the dose waste or undesirable, sometimes unexpected, harmful side effects. The structure and components of nanomaterials are the basic units for performing their corresponding functions, so we can expect that the elaborative regulation on the structure, component and other parameters, especially the ratio between different functional components (e. g. between therapeutic agents and adjuvant agents) of nanomedicines, will result in significantly improved therapeutic efficacy and biosafety.

Having addressed these challenges, and other issues currently less noticed, we believe that the strategy of nanomedicine-enabled chemotherapy in synergy with other treatment modalities can show ever-greater promise and bright prospects in the future of clinical cancer treatment.

## Data Availability

Not applicable.
